# Identification and Therapeutic Potential of a Vitronectin Binding Region of Meningococcal Msf

**DOI:** 10.1371/journal.pone.0124133

**Published:** 2015-03-31

**Authors:** Darryl J. Hill, Natalie J. Griffiths, Elena Borodina, Clio A. Andreae, Richard B. Sessions, Mumtaz Virji

**Affiliations:** 1 School of Cellular & Molecular Medicine, Medical Sciences Building, University Walk, University of Bristol, Bristol, BS8 ITD, United Kingdom; 2 School of Biochemistry, Medical Sciences Building, University Walk, University of Bristol, Bristol, BS8 ITD, United Kingdom; University of Helsinki, FINLAND

## Abstract

The human pathogen *Neisseria meningitides* (Nm) attains serum resistance via a number of mechanisms, one of which involves binding to the host complement regulator protein vitronectin. We have shown previously that the Meningococcal surface fibril (Msf), a trimeric autotransporter, binds to the activated form of vitronectin (aVn) to increase Nm survival in human serum. In this study, we aimed to identify the aVn-binding region of Msf to assess its potential as an antigen which can elicit antibodies that block aVn binding and/or possess bactericidal properties. Using several recombinant Msf fragments spanning its surface-exposed region, the smallest aVn-binding recombinants were found to span residues 1-86 and 39-124. The use of further deletion constructs and overlapping recombinant Msf fragments suggested that a region of Msf comprising residues 39-82 may be primarily important for aVn binding and that other regions may also be involved but to a lesser extent. Molecular modelling implicated K66 and K68, conserved in all available Msf sequences, to be involved in the interaction. Recombinant fragments which bound to aVn were able to reduce the survival advantage conveyed by aVn-interaction in serum bactericidal assays. Antibodies raised against one such fragment inhibited aVn binding to Msf. In addition, the antibodies enhanced specific killing of Msf-expressing Nm in a dose-dependent manner. Overall, this study identifies an aVn-binding region of Msf, an adhesin known to impart serum resistance properties to the pathogen; and shows that this region of Msf can elicit antibodies with dual properties which reduce pathogen survival within the host and thus has potential as a vaccine antigen.

## Introduction

*Neisseria meningitidis* (Nm) remains an important cause of meningitis and septicaemia worldwide. Case numbers vary geographically from 14 per 100,000 population across Europe compared to up to 1000 per 100,000 population during epidemics in sub-Saharan Africa [1]. Polysaccharide conjugate vaccines have now been developed to combat some serogroups of Nm e.g. serogroup C, a major problem in the UK and elsewhere and serogroup A responsible for African epidemics [2]. Due to the structural similarity between the serogroup B capsule and host glycans, polysaccharide vaccines are not effective against serogroup B Nm which accounts for the majority of meningococcal disease in developed countries. To overcome this limitation, surface proteins of Nm have been investigated as cross-protective vaccine components [3]. The likely effective protein candidates include those that enable meningococcal attachment to epithelial cells in their only niche, the human nasopharynx [4]; as it is from this site that Nm enters the blood and disseminates further in susceptible individuals, causing life threatening disease. However, once in the blood, Nm survival depends on its ability to avoid killing by the innate and adaptive immune defences of the host, and other likely vaccine candidates may include those bacterial molecules that aid such survival.

Polysaccharide capsule has long been known to be important for meningococcal haematogenous survival and spread [5]. In addition, a number of surface proteins such as factor H binding protein (fHbp), NspA and PorA have been shown to interact with regulators of the complement cascade including factor H [6,7] and C4 binding protein [8]. Binding of these regulators serves to interfere with the alternative and classical complement pathways respectively enabling Nm to resist complement killing for prolonged survival in the blood. Another key regulator of complement is vitronectin (Vn; previously known as S-protein), a multifunctional glycoprotein which protects bystander cells from complement-mediated cytolysis by inhibiting the insertion of the membrane attack complex (MAC, C5b-9) into cell membranes [9]. Vitronectin is a highly glycosylated protein of 459 amino acids and is produced by the liver. It exists in two forms 65kDa and 75kDa. Vitronectin can be found within the extracellular matrix or circulating within the blood stream [10]. In the blood, Vn protein circulates largely in its native folded monomeric conformation but can be activated (partially unfolded) by binding a range of physiological ligands conveying full functionality [10–13]. The N-terminus of Vn contains the somatomedin B domain [14,15] which is bound by plasminogen activator inhibitor type 1 (PAI-1) and the urokinase plasminogen activator receptor (uPAR; reviewed in [16]). The RGD motif responsible for mediating interaction with cell-expressed integrins is located at position 45–47 [17]. Vn also contains three heparin-binding regions (amino acids 82–137, 175–219 and 348–360 respectively) [18,19]. Through RGD and heparin binding domains of integrins, Vn is able to bind to and activate integrins such as α_v_β_3_ and α_v_β_5_ [15,20].

A number of bacterial species can bind to Vn and in doing so, are able to adhere to host cells via integrins and/or gain resistance to complement mediated killing (Reviewed in [21]). Previously, the integral outer membrane protein Opc of Nm has been shown to bind to the activated form of Vn (aVn) via sulphated tyrosines (Y56 and Y59) that become exposed on its unfolding and facilitate endothelial invasion [22,23]. In addition, Opc-Vn interactions protect Nm against complement-mediated killing by inhibition of the deposition of the terminal complement components C5b-9 (membrane attack complex, MAC) [24]. Another protein, Meningococcal surface fibril (Msf), a trimeric autotransporter, also leads to inhibition of MAC deposition on Nm and increased serum resistance [25].

Autotransporters (Type 5 secretion), are a protein superfamily of Gram negative bacteria, all possess an N-terminal signal sequence and a passenger domain of variable length which crosses the outer membrane via a C-terminal β-barrel forming domain [26]. In contrast to many monomeric autotransporters, the passenger domain of trimeric autotransporters often remains attached to the surface of bacterial cells where they are able to act as adhesins [27]. Trimeric autotransporters adhesins were first proposed to be a subfamily of autotransporters [28], but are now considered to be a distinct protein family of the autotransporter superfamily [27]. While the nomenclature of autotransporter for such proteins remains in use, we now know that a number of other proteins have roles to play in their surface presentation [29].

Our studies have shown that Msf allows meningococci to escape complement-mediated killing via binding to aVn [30] offering an explanation as to how this occurs. Thus *N*. *meningitidis* has two distinct means of binding to aVn. The main binding regions of Msf and Opc overlap and are located at the N-terminal region of Vn which contains the sulphated tyrosine residues essential for Opc binding. Msf, in contrast, does not require tyrosine sulphation for interaction with this region of Vn [22,30].

Msf was previously called Neisseria hia homologue A (NhhA) but also Hsf due to its homology with the *Haemophilus influenzae* (Hi) adhesins Hsf (Haemophilus surface fibril) and Hia (*Haemophilus influenzae* adhesin) [31,32]. Belonging to the trimeric autotransporter family of proteins, the haemophilus fibril is encoded by a single gene [33] and has been shown to mediate adhesion to epithelial cells via distinct binding domains (HsfBD1 and HsfBD2) which share homology with regions of Hia, a smaller autotransporter expressed by a quarter of non-typeable *H*. *influenzae* strains [28,34]. With a molecular weight of ~245kDa, Hsf is the largest autotransporter adhesin expressed by typeable *H*. *influenzae* and much larger than Msf (~60 kDa) [28,31]. Like Msf-mediated serum resistance demonstrated here for meningococci, Hsf binding to Vn conveys serum resistance to *H*. *influenzae* [30,35]. In the latter study, two Vn-binding regions were identified on Hi Hsf spanning amino acids 54–608 (554 residues) and 1536–2414 (878 residues) [35].

The ability of meningococci to target Vn via two distinct proteins suggests this interaction could aid survival *in vivo*, both at mucosal surfaces where Vn may be found [36] and in the blood. Targeting the processes by which meningococci are able to overcome complement-mediated killing in the blood may be a suitable strategy to eliminate disseminated disease caused by *N*. *meningitidis*. In the present study we demonstrate that the first 86 amino acids of Msf support binding to Vn. Our studies suggest amino acids 39–82 participate in Vn binding by molecular modelling. Antibodies against a recombinant Msf fragment spanning amino acids 1–203 block Vn binding to Msf and are able to mediate bactericidal killing of meningococci expressing Msf.

## Results

### Bioinformatic analyses of vitronectin binding domains within Msf

**Fig 1:**
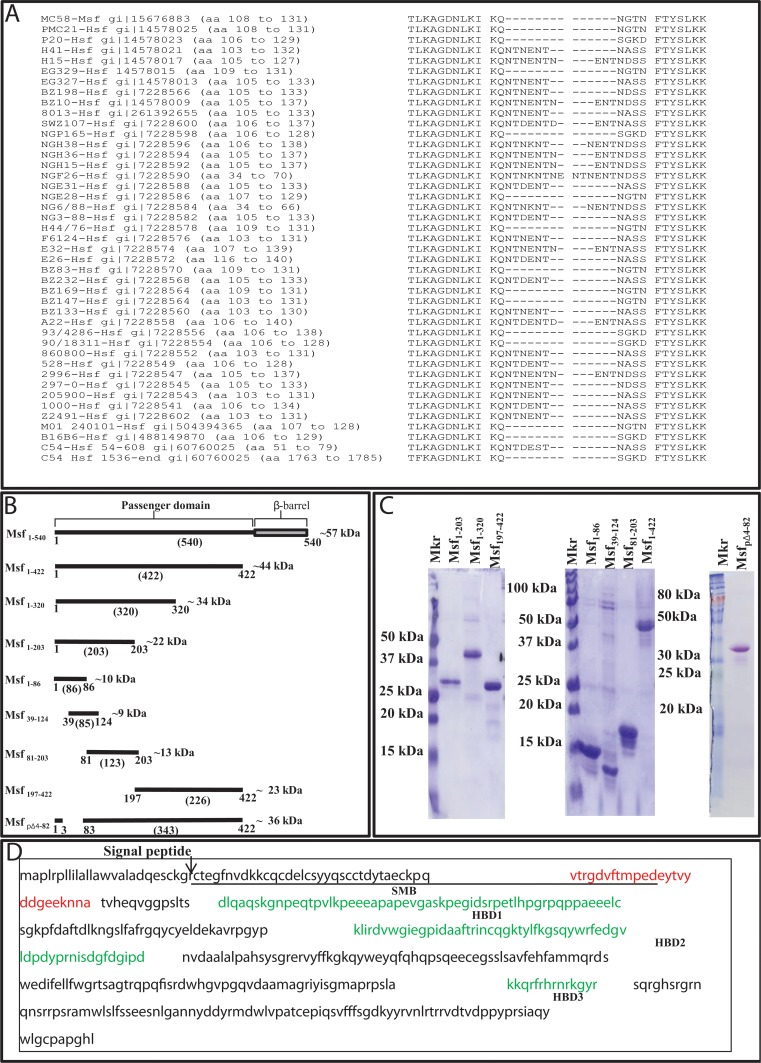
Regions of interest of meningococcal Msf and human vitronectin. A) Alignment of Msf of Nm and Hsf VB1 regions. Sequences were aligned by pairwise alignment using the ClustalW alignment method within BioEdit software. Strains and gi numbers are indicated at the left hand side of each sequence line. The numbering indicates amino acid position within the pre-protein in each individual sequence. B) Diagram showing the relative positions and theoretical molecular weights of recombinant Msf fragments generated and used in this study (excluding vector encoded amino acids). Full length mature Msf with the β-barrel is included for comparative purposes. The figures in parenthesis are the number of amino acids in each fragment excluding vector encoded amino acids. C) SDS-PAGE of the recombinant Msf fragments produced in this study. In each instance, 2 μg of protein was loaded and run on 15% SDS-PAGE gels which were stained with Coomassie blue. D) Sequence of human vitronectin and of the Msf-binding region. The sequence of full length human vitronectin is shown (Accession number CH471159.1) indicating the position of the Msf binding peptide VA-26 spanning amino acids 43–68 of the mature peptide (red). Residues spanning the somatomedin B (SMB) domain are underlined in black [14,15]. The following residues (~53–130) comprise the connecting region of Vn. Three heparin binding regions identified in vitronectin (HBD1-3) are shown in green [18,19].

We have previously demonstrated that Msf binds to Vn conveying serum resistance to meningococci [30]. In the studies of Hallstrom et al [35], on the interactions of *H*. *influenzae* Hsf with Vn, two independent Vn binding regions were identified, spanning amino acids 54–608 (544 residues) and 1536–2414 (878 residues). Hsf is expressed by typeable *H*. *influenzae*, whilst a proportion (~25%) of non-typeable *H*. *influenzae* strains express the closely related Hia which is ~1200 amino acids in length. Meningococcal Msf is the smallest of the three related molecules at ~590 amino acids prior to cleavage of the signal peptide. Of note, both of the recombinant Hsf proteins shown to bind to Vn are larger than the mature Msf protein of ~540 amino acids. In order to identify potential Vn binding regions of Msf, we aligned both regions of Hsf containing Vn binding properties against Msf sequences from the NCBI database. Alignment revealed a number of regions with high percentage identities (Table 1). Notably one region (Msf-VB1) was similar between both Vn binding regions of Hsf and Msf (Fig 1A).

**Table 1:** Putative vitronectin binding regions within Msf identified by alignment against Hsf.

Name	Sequence	Amino acid range of mature H44/76 Msf
VB1	TLKAGDNLKIKQNGTNFTYSLKK	58–80
VB2	TEKLSFSANGNKVNITSDTKGLNFAK	91–115
VB3	VHLNGIGSTLTD	126–137
VB4	AASVKDVLNAGWNIKG	160–176
VB5	NGKKTEVKIGAKTSVIKEKDGKLVTGK	215–241
VB6	TDEGEGLVTAKEVIDAVNKAGWRMKTTTANGQ	250–281
VB7	FETVTSGTNVTFASGKGTTATVSKDDQGNITVMYDVNVGDAL	288–329

### Generation and vitronectin-binding properties of recombinant Msf fragments

In order to test if Msf-VB1 or other region(s) of Msf bind to Vn, Msf recombinant fragments were produced spanning the length of Msf based on the sequence from strain H44/76. A schematic overview of recombinant molecules produced is shown in Fig 1B and the purified products characterised by SDS-PAGE (Fig 1C) and Western blotting (not shown).

To identify which region of Msf facilitated binding to Vn, throughout this study we have used the activated form of vitronectin (aVn) following our previous investigation which established that Msf requires the activation of Vn for efficient binding. Such activation may be achieved by denaturation using heat or urea for example, or by activation of its monomeric form on coating to ELISA plates as described previously [30].

**Fig 2:**
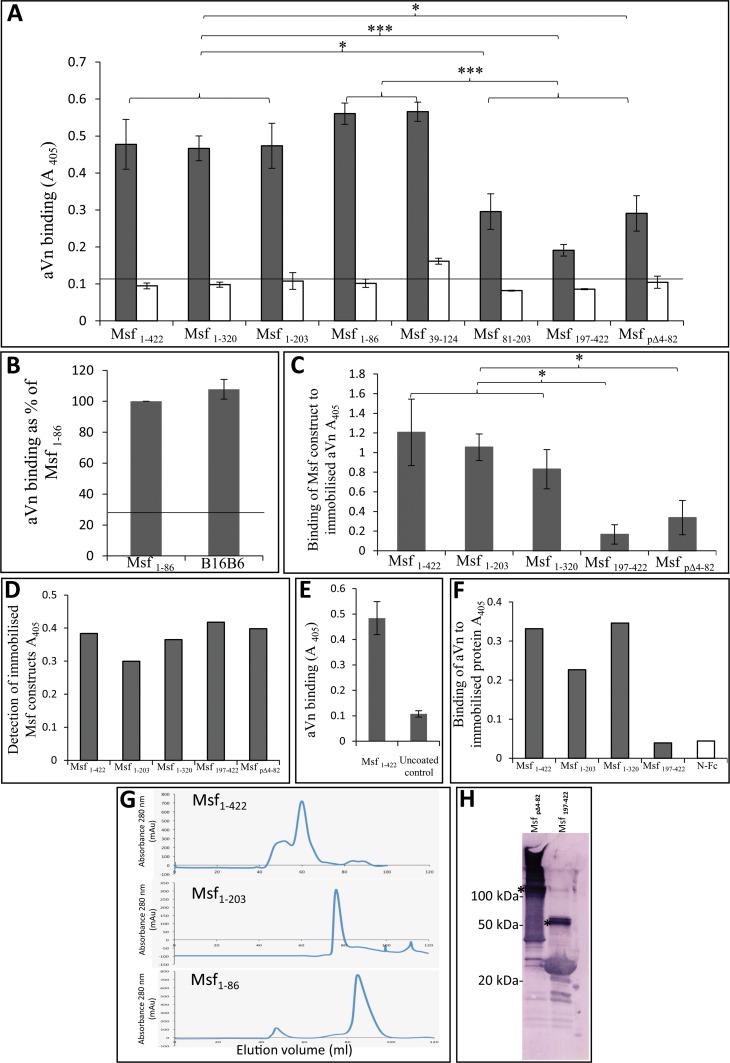
Binding of aVn to recombinant Msf constructs. A) ELISA plates were coated with Msf constructs (3 μM) and overlaid with aVn (74 nM). Bound aVn was detected using polyclonal rabbit anti-vitronectin antibody followed by alkaline phosphatase conjugated anti-rabbit antibody (grey columns). In control experiments, aVn was omitted (white columns). Each experiment was performed three times incorporating triplicate determinations within each experiment. Overall means (*n* = 3) and SD are shown. Msf_1–422_, Msf_1–320_, Msf_1–203_, Msf_1–86_ and Msf_39–124_ were found to bind similar and significantly higher levels of aVn compared to Msf_197–422_, Msf_pΔ4–82_ or Msf_81–203_. *P<0.05, ***P<0.0001 as indicated. B) ELISA plate coated with H44/76 Msf_1–86_ and the equivalent region from Strain B16B6 and overlaid as A) above. No significant difference in binding between Msf from the two strains was observed (*n* = 3 ± SE are shown). C) Binding of recombinant Msf constructs to aVn. ELISA plates coated with aVn (74 nM) were overlaid with the Msf constructs (3 μM). Bound recombinant Msf was detected using rabbit anti-Msf polyclonal antiserum followed by alkaline phosphatase conjugated anti-rabbit antibody. Msf_1–422_, Msf_1–203_ and Msf_1–320_ bound at similar levels to immobilised aVn compared with Msf_197–422_ which was approximately 6 fold lower compared with Msf_1–422_. The Δ4–82 derivative of Msf_1–422_ also lost aVn binding capacity which was reduced by ~4-fold. Mean values of three independent experiments ± SD are shown. *P<0.05. D) Control experiment to establish equivalent detection of Msf constructs using anti-Msf polyclonal antibody. Recombinant Msf constructs coated onto the ELISA plate (3μM) were overlaid with anti-Msf polyclonal antibody and anti-rabbit alkaline phosphatase conjugated secondary antibody. Plates were subsequently developed with pNPP substrate (Sigma). Data shown are means of triplicate determinations within a single experiment. E) ELISA wells were either coated with Msf_1–422_ (3 μM) or uncoated prior to blocking. Wells were overlaid with polyclonal rabbit anti-vitronectin followed by alkaline phosphatase conjugated anti-rabbit antibody prior to developing. Means of three independent experiments ± SE are shown. F) ELISA plates were coated with recombinant Msf proteins (3μM), overlaid aVn (74nM) and bound aVn detected with anti-vitronectin polyclonal antibody and anti-rabbit alkaline phosphatase conjugated secondary antibody (grey columns). An unrelated protein (CEACAM1 N-Fc) was used in the experiment as a control to determine nonspecific aVn binding (white column). Data shown are means of triplicate determinations within a single experiment. G) Size exclusion chromatograms prepared using an S200 column for Msf_1–422_, _1–203_ and _1–86_ as indicated. Peak elution volumes and predicted molecular weights were obtained as follows Msf_1–422_ 59.3 ml and 270 kDa, Msf_1–203_ 76.1 ml and 67 kDa and Msf_1–86_ 84.1 ml and 34.5 kDa indicative of oligomeric forms of each construct. H) Western blot of unheated Msf recombinant proteins (as indicated) following SDS-PAGE. Msf was detected in each instance using anti-Msf polyclonal antibody raised in rabbit followed by anti-rabbit-alkaline phosphatase conjugated antibody prior to chromogenic development. Notably oligomeric bands were detected in the region approximating a trimeric molecular weight for each protein (*). In panel A and B the black line indicates the level of aVn binding observed to the unrelated control protein (Fig 2F).

We examined the binding of aVn to recombinant fragments within the Msf passenger domain using ELISA. Immobilised Msf fragments were overlaid with aVn which was detected using anti-Vn polyclonal antibody and appropriate secondary antibody (Fig 2A). Vitronectin bound to the recombinant Msf containing residues 1–422 and similar aVn binding levels were observed when using Msf constructs Msf_1–422_, Msf_1–320_, or Msf_1–203_. However, comparatively less binding was observed to Msf_197–422_. A recombinant Msf_1–422_ construct was produced lacking amino acids 4–82 (Msf_pΔ4–82_; Fig 1B). Deletion of this region resulted in decreased aVn binding compared with the Msf_1–422_ protein (Fig 2A). Overall these data suggest the Vn binding involves the region between amino acids 4 and 82 of the mature Msf passenger domain. In order to refine further the aVn binding region of Msf, smaller recombinants spanning Msf_1–203_ were produced i.e. Msf_1–86_, Msf_39–124_ and Msf_81–203_ (Fig 1A). Of these proteins, Msf_1–86_ and Msf_39–124_ bound similar and higher levels of aVn than Msf_81–203_ (Fig 2A).

Collectively these data provide evidence that aVn binding occurs predominantly between amino acids 39–82 of Msf correlating with the predicted VB1 region (Msf_58–80_) identified as present in Msf and both Vn binding regions of Hsf (Table 1). In addition, the data suggest other regions of Msf may also bind to aVn.

### Diversity amongst the aVn binding regions of Msf

A comparison of Msf protein sequences within the NCBI database revealed identities of 84.9–100%. Despite some diversity between the Msf binding region from different strains, Msf from Strains H44/76 and strain B16B6 (two of the most diverse Msf sequences) have previously been shown to bind equally well to aVn [30]. Accordingly, B16B6 Msf construct Msf 1–86 also bound equally well to aVn (Fig 2B). Hence Msf and the aVn binding function appear to be highly conserved between different meningococcal strains.

The ELISA data shown in Fig 2A were obtained using immobilised Msf overlaid with aVn. Controls and additional experiments confirmed the specific nature of aVn interaction with the Msf constructs. A similar binding pattern to Fig 2A was observed if immobilised aVn was overlaid with recombinant Msf and detected with anti-Msf polyclonal antibody (Fig 2C (Fig 2D shows an Msf construct detection control)). Anti-vitronectin and secondary antibody controls gave comparably low absorbance values to coated and uncoated wells (Fig 2A and 2E). Further, aVn binding to an unrelated control protein (N-Fc, shown in Fig 2F) was at a level comparable to the direct binding of anti-Vn to Msf constructs in the absence of aVn (blank columns, Fig 2A) i.e. the general low level background colour development observed in several ELISA experiments was comparable (Fig 2A, 2D and 2F).

Msf would normally be expressed by meningococcal cells in a trimeric form [30]. Whilst we do not know if trimerisation is a prerequisite for Vn binding, it is possible that a lack of trimerisation by recombinant proteins could influence Vn binding. We were able to show by gel filtration column that Msf_1–422_, _1–203_ and _1–86_ and by Western blotting that Msf_197–422_ and Msf_pΔ4–82_ were able to form trimers (Fig 2G and 2H).

### The binding of Msf constructs to the peptide spanning residues 43–68 (VA-26) of human vitronectin

**Fig 3:**
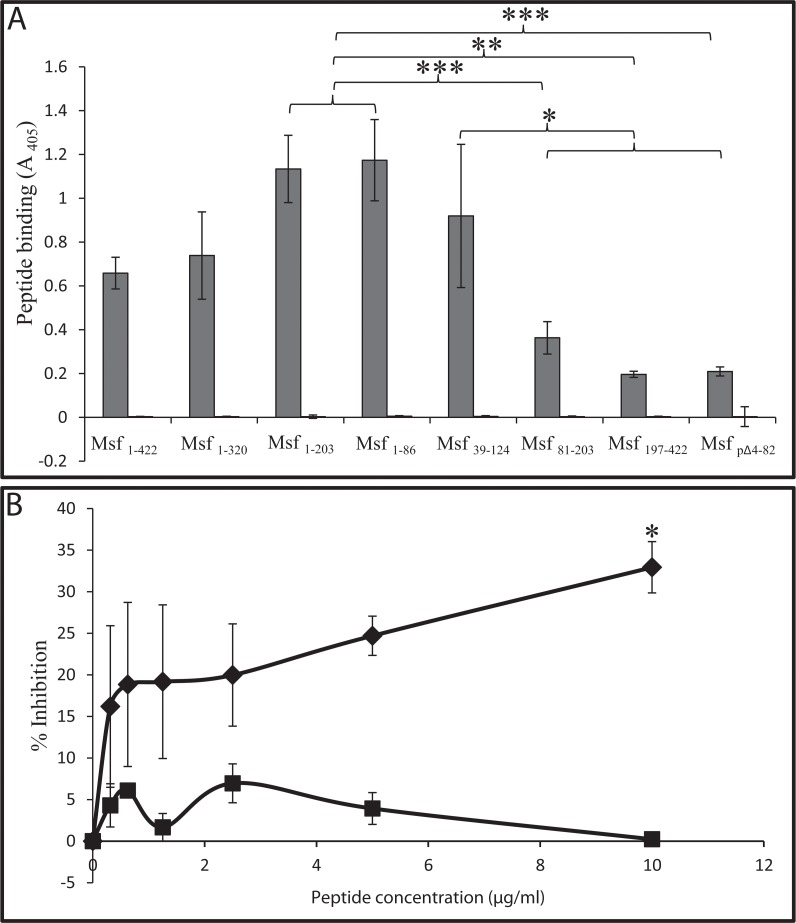
Binding of vitronectin peptide VA-26 to recombinant Msf constructs. A) ELISA plates coated with Msf constructs (3 μM) shown were overlaid with biotinylated VA-26 (1.5 μM; grey bars) or biotinylated control peptide (white bars). In each instance, bound peptide was detected using alkaline phosphatase conjugated anti-biotin antibody. No binding of the biotinylated control peptides (MV-14 or GS-22) was observed to any Msf construct, whereas constructs, Msf_1–203_, Msf_1–86_ and Msf_39–124_ bound significantly more VA-26 compared with Msf_197–422_, Msf_pΔ4–82_ or Msf_81–203_ in keeping with the observations with aVn (Fig 2). Means of three independent experiments ±SD are shown. * *P*<0.05, ** *P*<0.001 and *** *P*<0.0001 as indicated. B) ELISA plates were coated with Msf_1–422_ (3 μM) and overlaid with aVn (74 nM) in the presence of either VA26 (black diamonds) or a control peptide (black squares). Bound aVn was detected using polyclonal rabbit anti-vitronectin antibody followed by alkaline phosphatase conjugated anti-rabbit antibody. Each experiment was performed three times incorporating triplicate determinations within each experiment. Overall means (*n* = 3) and SE are shown. *P<0.05.

We previously reported that recombinant Msf bound to a region corresponding to the amino acids 43–68 of human Vn shown in Fig 1 and [30]. To test the binding of Msf fragments to this region of Vn, a biotinylated peptide spanning human Vn residues 43–68 (VA-26: VTRGDVFTMPEDEYTVYDDGEEKNNA) and a control peptide were applied to ELISA plates coated with recombinant Msf fragments. Vitronectin peptide binding was detected using an anti-biotin alkaline phosphatase conjugated antibody. The interactions occurred specifically with VA-26 and mirrored those observed with whole aVn; the best binding was observed to fragments containing amino acids 39–82 (Fig 3A). Thus overall the data support binding of amino acids 43–68 of mature human Vn with amino acids 39–82 of neisserial Msf. However, low level binding of Msf_197–422_, Msf_pΔ4–82,_ and Msf_81–203_ to VA-26 but not to the control peptide (Fig 3A) suggests Msf regions other than 39–82 may also interact with Vn residues 43–68. To further investigate the specificity of the interaction between Msf and aVn, we demonstrated that VA26 was able to inhibit the binding of aVn to immobilised Msf_1–422_ (Fig 3B).

### Specific Msf amino acids involved in vitronectin binding

**Fig 4:**
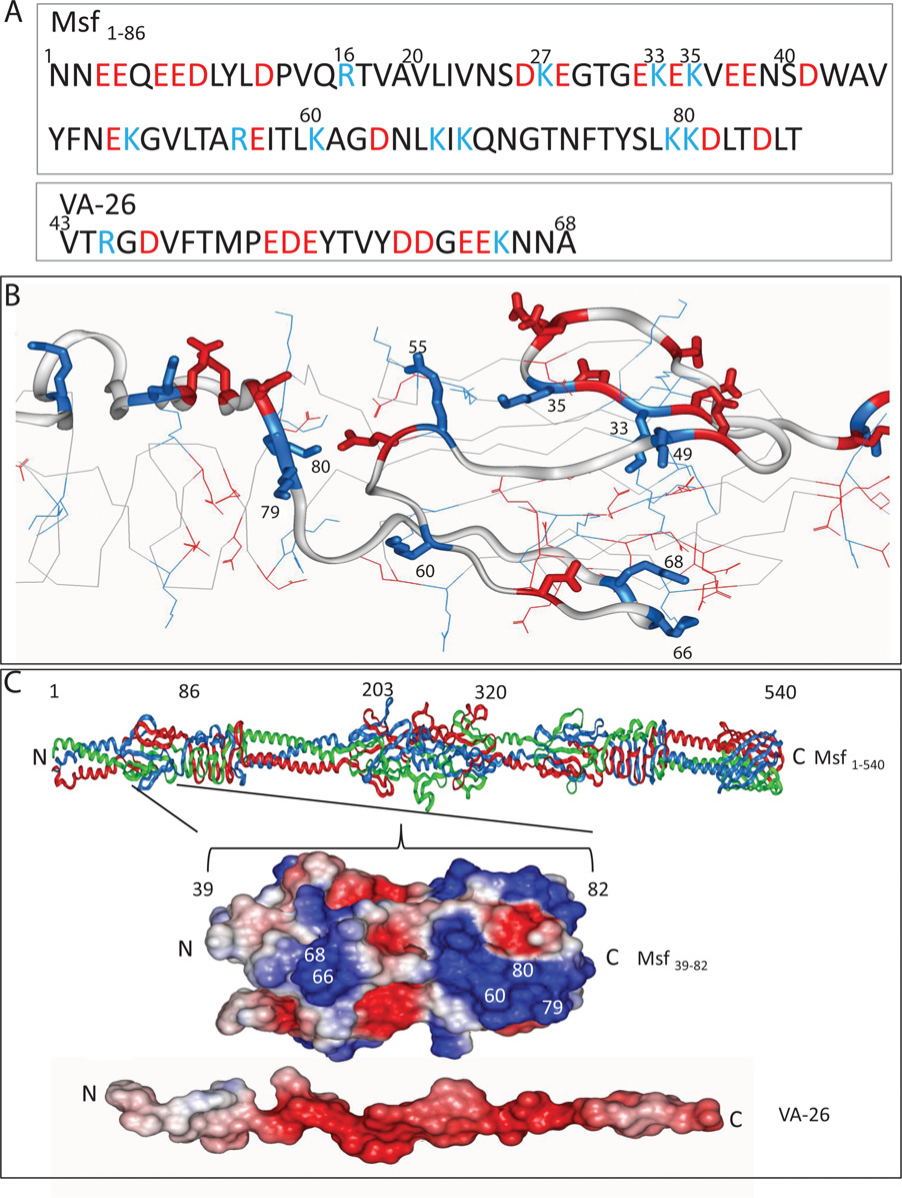
Charge distribution and molecular modelling of Msf_1–86_ and VA-26. A) Charge distribution of Msf_1–86_ and VA-26. B) Molecular model of the N-terminal region of Msf showing side chains of the charged residues and the backbone (C-alpha trace). C) Ribbon structure of mature Msf is presented (top) to show the position of the vitronectin binding region of Msf (Msf_39–82_). The electrostatic surface of the Msf_39–82_ (middle) with the VA-26 peptide (bottom) in a β-strand extended conformation, illustrating the charge complementarity between this highly positively charged region of Msf and the highly negatively charged VA-26 peptide. The positively charged residues are shown in blue and the negative residues are shown in red in A-C. Amino acids chosen for alanine substitution by site directed mutagenesis are shown in white on a single strand of the Msf trimer (C middle).

To identify the Msf residues within the region 39–82 that engage with the aVn peptide VA-26, a charge analysis of Msf_1–86_ and VA-26 was conducted (Fig 4A). A relatively positively charged patch in Msf_1–86_ was identified spanning amino acids 55–80 of mature Msf. This region correlates with the sequence of theVB1 region of H44/76 (Table 1) and lies within the 39–82 residue stretch proposed as a predominant Vn binding region from the data shown in Fig 4. In order to test the hypothesis that basic residues within this region interact with the acidic region of Vn represented in VA-26 peptide (Fig 4A), we generated a model encompassing Msf_1–86_ (Fig 4B) in order to identify surface exposed amino acids. The electrostatic charge of the models at pH 7 was mapped onto the surfaces of Msf 39–82 and VA-26 showing charge complementarity between the two (Fig 4C). Despite Msf being a trimer, several loops with linear stretches of surface exposed amino acid residues were observed in the model. Based on this model, the basic residues K60, K66, K68, K79 and K80 are surface exposed (Fig 4C) and also conserved within all Msf sequences available in the NCBI database. Other basic residues in close proximity within Msf_1–86_ (e.g. K33, K35, K49 and R55) were not conserved amongst Msf from different Nm isolates. A strategy was devised to introduce three sets of mutations into Msf_1–203_ (K60A, KIK66-68AIA and KK79-80AA) based on conservation of the amino acid, primer suitability and minimal disruption to the predicted structure.

### Vitronectin binding to Msf mutants

**Fig 5:**
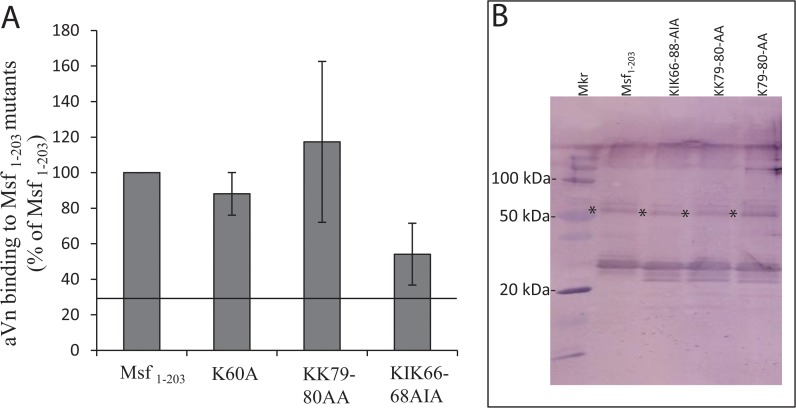
Binding of aVn to site directed mutants of recombinant Msf. A) ELISA plates coated with Msf constructs shown were overlaid with aVn and the bound aVn was detected using rabbit anti-vitronectin polyclonal antibody followed by an alkaline phosphatase conjugated anti-rabbit antibody. No Significantly different aVn binding was observed between Msf_1–203_ and the other constructs. Means of three independent experiments and standard deviations are shown. The black line indicates the level of aVn binding observed to an unrelated protein (Fig 2F). B) Western blot of Msf recombinant proteins (as indicated) following SDS-PAGE where samples were not heated. Msf was detected in each instance using anti-Msf polyclonal antibody raised in rabbit followed by anti-rabbit-alkaline phosphatase conjugated antibody prior to chromogenic development. Notably oligomeric bands were detected in the region approximating a trimeric molecular weight for each protein (*).

In ELISA using immobilised Msf_1–203_ or its mutant derivatives, no significant effect of either K60A or KK79-80AA mutation on Vn binding was observed (Fig 5A). The double mutant KIK66-68AIA showed a reduction in Vn binding however was not statistically significant in keeping with the other mutants (Fig 5A). Further, a reduction in Vn binding of ~45% suggests the involvement of residues other than or including K66 and K68. Further mutational analyses and other studies such as co-crystallisation would be required to identify the precise molecular nature of Msf-aVn interface. All Msf recombinants with mutations retained the capacity to trimerise (Fig 5B).

### Blocking of vitronectin binding by anti-Msf antibody

**Fig 6:**
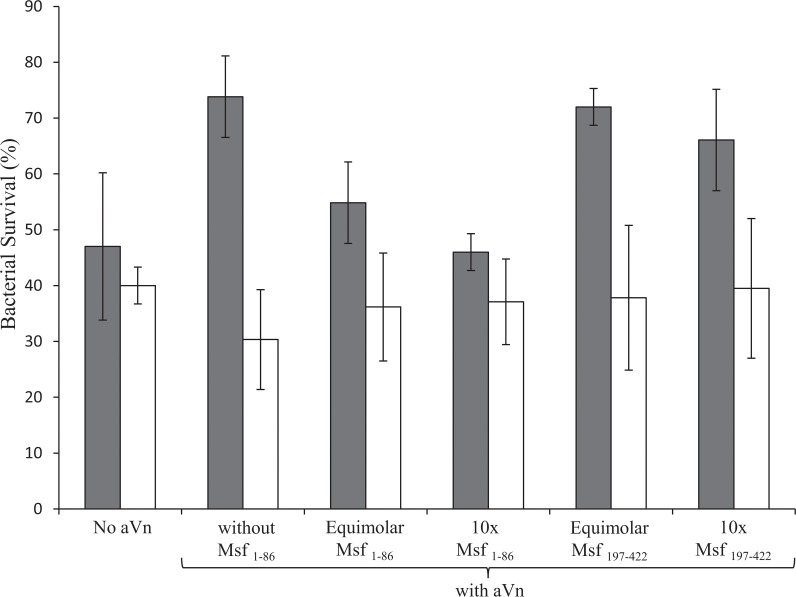
Inhibition of aVn-mediated increased serum resistance by vitronectin-binding Msf constructs. Serum bactericidal assay using MC58 Msf+ *Δopc* (grey bars) MC58 *Δmsf Δopc* (white bars). Bacteria were incubated in normal human serum (10%) with aVn (10 μg/ml) or without aVn as indicated. Where included, Msf fragments were added at either equimolar or 10x excess molar amounts relative to aVn as indicated. In the presence of Msf_1–86_ but not Msf_197–422_ the protective effect of aVn was diminished. Data shown are average values and ranges from two independent experiments each comprising triplicate determinations.

We have shown previously that the binding of Vn by Msf conveys a survival advantage to meningococci through increased resistance to serum killing [30]. Thus introduction of aVn-binding Msf fragments into serum bactericidal assays should reduce the survival advantage of Msf-expressing meningococci. Indeed Msf_1–86_ but not Msf_197–422_ reduced aVn-mediated increased serum resistance of Nm (Fig 6). This observation also suggested that such peptides may generate antibodies capable of blocking Vn interactions though binding to Msf on the surface of meningococci. Further, as anti-Msf antibodies have been reported to be bactericidal [32], antibodies directed against the Vn-binding domain of Msf may be bactericidal in addition to blocking Vn binding.

**Fig 7:**
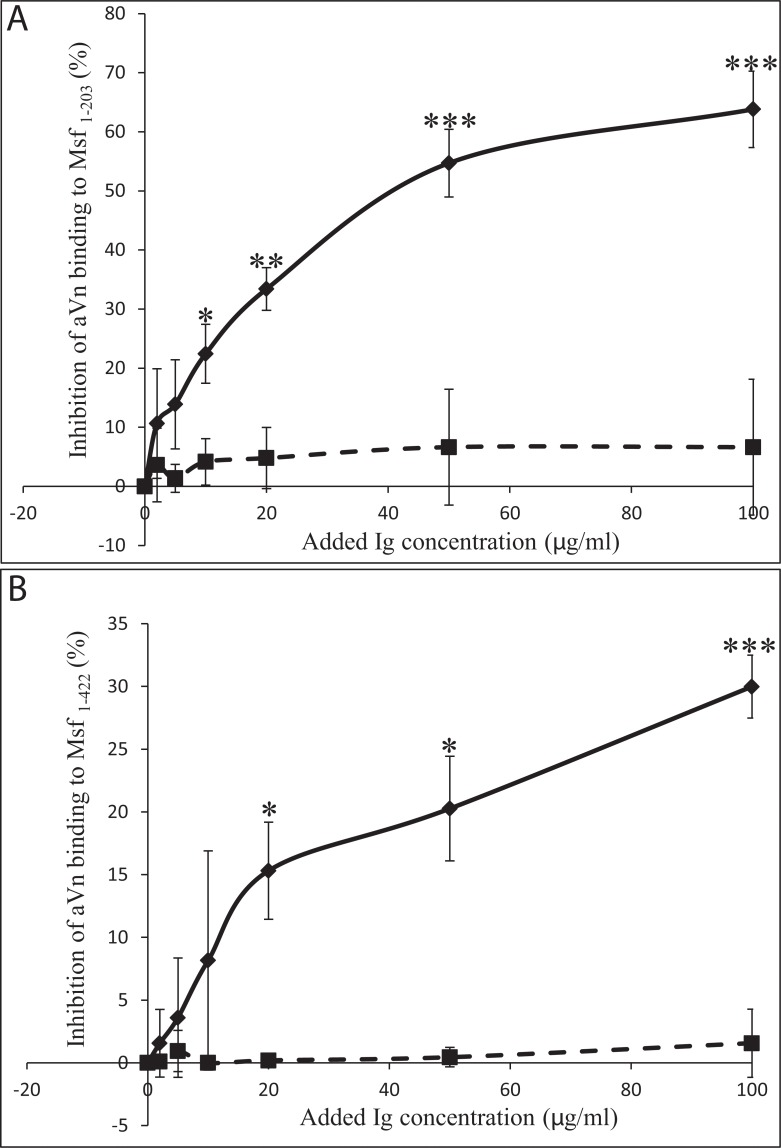
Inhibition of aVn binding by anti-Msf_1–203_ antibodies. ELISA plates coated with Msf_1–203_ (A) or Msf_1–422_ (B) were overlaid with aVn (74 nM) in the presence of purified anti-Msf_1–203_ (solid lines) or anti-CEACAM rabbit polyclonal antibody control (dashed lines). Anti-Msf_1–203_ inhibited aVn binding to both Msf proteins in a dose dependent manner. Data shown are means of three independent experiments ± SD. * *P*<0.05, ** *P*<0.001 and *** *P*<0.0001 as indicated.

In order to determine if anti-Msf_1–203_ antibodies could block aVn binding, rabbit antiserum against Msf_1–203_ was generated and affinity purified antibodies used in inhibition ELISAs. A dose-dependent inhibition of aVn binding to Msf_1–203_ and Msf_1–422_ was observed in the presence of anti-Msf_1–203_ but not the control antibody (A0115 purified rabbit anti-CEACAM polyclonal antibody; Fig 7). Inhibition of Vn binding was much more pronounced for Msf_1–203_ than Msf_1–422_. The data support the hypothesis that whilst Vn binding predominates within Msf amino acids 39–82, other regions of Msf may also interact with Vn. An alternative explanation is that structures of Msf outside amino acids 1–203 constrain the access of the antibodies in some way. Further studies are required to clarify these points.

### Serum bactericidal assay

**Fig 8:**
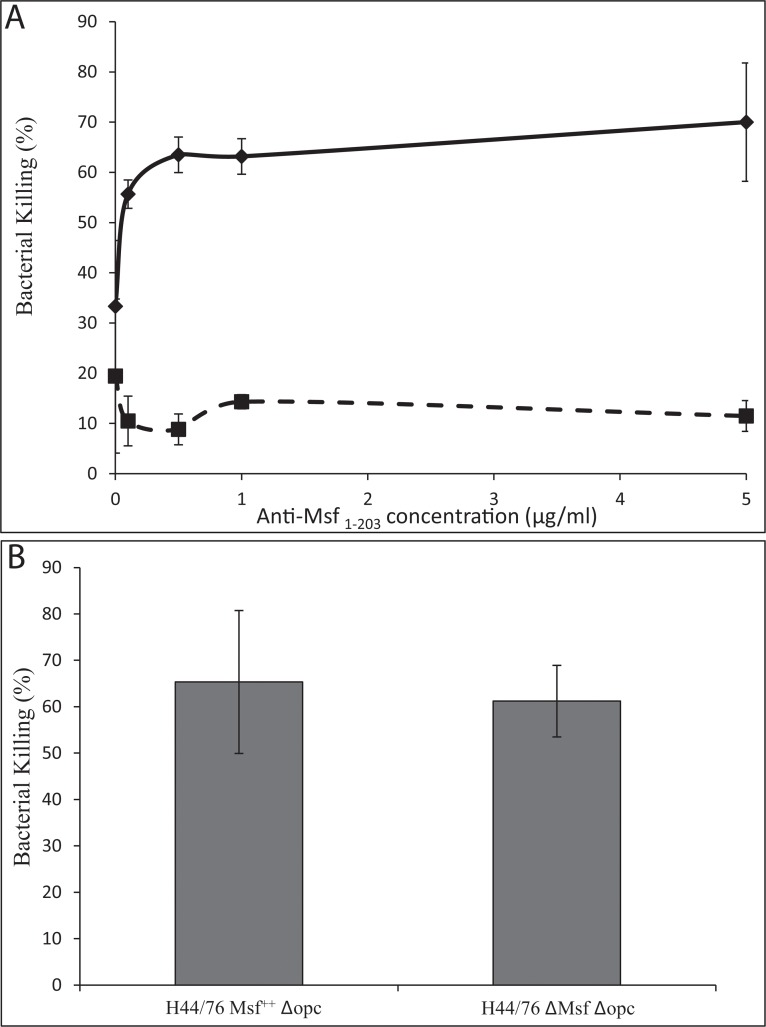
Serum bactericidal activity of anti-Msf_1–203_ antibodies. A) H44/76 Msf^++^ *Δopc* and its *Δmsf Δopc* mutant were incubated in the presence of varying concentrations of purified rabbit anti-Msf_1–203_ as described in methods. A dose dependent increase in killing of H44/76 Msf^++^ *Δopc* (solid line) but not its *Δmsf* mutant (dashed line) was observed. Two independent experiments were carried out incorporating triplicate determinations. Ranges and average values of the means from these experiments are shown. B) In a control experiment the Nm derivatives were incubated with rabbit antiserum raised against bacterial cell lysates. Both strains were equally susceptible to killing in the presence of rabbit anti-Nm polyclonal antiserum. For B) Means and standard deviations are shown *n* = 3.

In serum killing experiments using 10% human serum with increasing concentrations of purified anti-Msf_1–203_ antibodies, killing of Msf-expressing Nm strain H44/76 Msf^++^ *Δopc* compared to the Δmsf mutant were observed at all concentrations of antibody used (Fig 8A). In control experiments, both H44/76 Msf^++^ *Δopc* and H44/76 *Δmsf Δopc* were killed equally well by rabbit polyclonal anti-meningococcal antiserum (Fig 8B). These data support the notion that meningococcal killing in this instance is specifically mediated by anti-Msf antibodies directed against Msf on the bacterial surface and specifically the Msf regions involved in binding to aVn.

## Discussion

Here we provide evidence that the main aVn binding domain of Msf is located between amino acids 39–82 of the mature protein. A recombinant Msf fragment containing this region generated antibodies that were capable of blocking Msf interaction with Vn and elicited a dose-dependent serum bactericidal response. Many bacterial species such as *N*. *meningitidis*, *N*. *gonorrhoeae*, *H*. *influenzae*, *Streptococcus pneumoniae* and *Moraxella catarrhalis* express surface proteins that bind to Vn and are able to adhere to and invade epithelial and endothelial cells via Vn-binding cell surface integrins [23,37–41]. In addition, several of these proteins, via binding to Vn, increase bacterial resistance to killing in human serum. The latter include Hsf, Protein E and Protein F of *H*. *influenzae* [35,42,43], Opc and Msf of *N*. *meningitidis* [22,23,30] and UspA2 of *Moraxella catarrhalis* [44]. In the case of the trimeric autotransporter adhesin UspA2, mutation leading to a loss of UspA2 expression leads to a loss of both Vn binding and decreased survival in human serum [44]. Vitronectin binding has been reported to lie within amino acids 30 and 170 of the 630 residue UspA2 protein used in the study [45]. However, not all UspA2 proteins bind to Vn or convey serum resistance [46,47]. UspA2 was shown to bind to the heparin binding region located in the C-terminal area of Vn involving a region of UspA2 that is antigenically variable [46] and thus may not be suitable for use as a vaccine antigen. In contrast, meningococcal Vn-binding Msf is relatively conserved and those proteins with the greatest sequence diversity are able to bind to Vn (this study and [30]).

Hsf of *H*. *influenzae*, like UspA2, is a trimeric autotransporter adhesin [28]. Two regions or the protein spanning amino acids 54–608 (554 residues) and 1536–2414 (878 residues) have been implicated in binding to Vn [35]. Both of these regions are larger than the mature Msf protein of ~540 residues [31]. Vitronectin binds to recombinant Msf fragment containing amino acids 1–86, notably this recombinant protein contains a sequence present in both of the Vn binding regions of Hsf. We also showed that this region of Msf bound to a peptide spanning amino acids 43–68 (VA-26) of Vn. Both Msf and Opc also bind to the heparin binding region of Vn albeit via different mechanisms [22,30]. Akin to Opc but not Msf, Hsf has previously been shown to bind to Vn in a heparin dependent manner [35]. Whether Hsf, or the related Hia, bind to the connecting region of Vn in similar fashion to Msf remains to be determined. Model data presented in the current study indicate that amino acids K66 and K68 may have a role in Msf binding to Vn and notably both residues are conserved in Msf and also Hsf and Hia sequences.

As illustrated above, in common with *N*. *meningitidis*, both typeable and non-typeable *H*. *influenzae* strains are able to express multiple Vn binding proteins and acquire serum resistance. The evolution of such multiple means of recruiting Vn in the respiratory pathogens suggests that this interaction could be of particular importance in their survival in the host and may have potential as vaccine antigens. Serum resistance by both of these human pathogens is acquired via a number of other means also, capsule expression being the major feature imparting this property. In Nm, strains expressing serogroup B capsule in particular account for ~80% of invasive meningococcal disease in the UK, North America, Australasia and regions of South America [48], whereas other serogroups predominate in specific geographic locations notably serogroup A is associated with epidemics in sub-Saharan Africa [1]. The introduction of vaccines against capsular antigens has led to an overall reduction in serogroups A, C, Y and W-135 where the vaccines have been introduced. Unlike other serogroups, group B capsular antigen is generally not considered suitable as a vaccine antigen due to its similarity to glycans presented on human N-CAM. The development of vaccines based on non-capsular antigens is therefore required and could offer protection against not only serogroup B, but all meningococcal serogroups. Notably, two licensed vaccines contain factor H binding protein (fHbp), which binds to the complement regulator factor H [49,50]. Studies examining the potential coverage of one of these vaccines (Bexsero) suggest approximate coverage of 73% of isolates in the UK and 66% of isolates in Canada where in both countries serogroup B accounts for the majority of invasive meningococcal disease [51,52]. Therefore, there is still a need for identification of additional antigens to improve upon meningococcal strain coverage to ensure complete eradication of meningococcal disease. In considering other antigens, notably, the ability of Msf to induce bactericidal antibodies has been shown in previous studies suggesting its potential as a vaccine candidate [32]. Further, Msf expression has been shown to be upregulated under conditions that mimic different human niches, for example on epithelial attachment [25] and in response to changes in iron presentation [53]. These observations suggest that Msf expression may be higher in *in vivo* organisms than observed in those grown *in vitro* and thus imply its importance for survival in the host. For other Nm adhesins such as factor H binding protein, concerns have been raised relating to whether the presence of functional regions in the vaccine antigen, through its masking by recruitment of factor H following immunisation, could limit bactericidal response against it [54]. Such studies are difficult to evaluate in the case of factor H binding protein as its binding is specific for human factor H. This is not the case for Msf, as Msf can bind to other vitronectins (e.g. mouse, bovine and rabbit) besides human Vn [30]. In the current study, we present data which show that a recombinant vitronectin-binding region of Msf can elicit antibodies in species (rabbit) whose vitronectin can bind the protein. Although we have used peptides which are larger than the main Vn-binding domain of Msf, these antibodies have both function-blocking (inhibiting aVn binding) and bactericidal effects. In conclusion, through the present studies, we provide evidence that the use of peptides that contain functional regions of some bacterial complement regulator-binding proteins can offer a viable approach as a vaccine strategy. Further detailed evaluation is required to assess if Msf can indeed serve as an additional candidate for prevention of meningococcal disease.

## Materials and Methods

### Ethics statement

Human serum was used as a source of endogenous complement and has been described previously [30]. Sera were obtained from the National Blood Service, Bristol or derived from blood collected from healthy adult volunteers; written approval was obtained in each case. The collection of blood and the research described complies with the relevant guidelines and institutional practices (University of Bristol Hospital Trust Local Research Ethical Committee E4388). No animals were used in this study. However, animal products used were purchased from commercial suppliers and were produced conforming to appropriate national guidelines.

### Neisserial strains

Strains expressing Msf, but lacking Opc expression, namely MC58 Msf^+^ *Δopc* and H44/76 Msf^++^ *Δopc* or the double mutants, MC58 *Δmsf Δopc* and H44/76 *Δmsf Δopc*, lacking both the Vn binding proteins, have been described in previous studies [30]. All neisserial strains were grown on HBHI agar [brain-heart infusion agar (LabM) supplemented with heated horse blood (TCS Biosciences) as described previously [55].

### Sequence alignments

Sequence alignments were made using the Clustal W algorithm of BioEdit software.

### Purified proteins and synthetic peptides

Human Vn in its unfolded, activated form (aVn; purified by urea treatment and heparin-sepharose chromatography) was purchased from Sigma. The biotinylated synthetic peptide spanning the Vn residues 43–68 (VA-26) was obtained from GL Biochem (Shanghai, China) and the biotinylated control peptide MV-14, with scrambled sequence of residues spanning the Vn region 41–54, was synthesised by Pepceuticals (Nottingham, UK). The unrelated biotinylated control peptide GS-22 was a kind gift from Dr Graham Bloomberg; University of Bristol).

### Antibodies

Vitronectin binding to recombinant Msf was determined using a rabbit polyclonal anti-vitronectin antibody (AB19014, Millipore). Msf recombinant regions were detected using rabbit polyclonal antiserum (TA-40) described previously [30]. Anti-Msf_1–203_ antiserum was provided by ThermoScientific. Purified anti-Msf antibodies were obtained by affinity chromatography using recombinant Msf_1–203_ conjugated to an Aminolink plus column according to the manufacturer’s instructions (Pierce).

### Generation of recombinant Msf regions

Production of the His-tagged passenger domain of Msf was as described previously [30]. Regions of *msf* were amplified using primers shown in Table 2. PCR products were *Nde*I /*Xho*I cloned into pET24b, expressed in *E*. *coli* (BL21 (DE3)) prior to purification under native conditions. Briefly, *E*. *coli* were grown to OD_600_ 0.5 in LB both and recombinant Msf region expression was effected by the addition of 1 mM IPTG, 3 h, 37°C. Cells (from 500 ml LB broth) were harvested by centrifugation (6,000 *g*, 15 min, 4°C) and resuspended in 60 ml 50 mM Tris (pH 7.5), containing 250 mM NaCl and 10 mM imidazole. Cells were disrupted by sonication on ice, insoluble material was pelleted by centrifugation (10,000 *g* for 25 min at 4°C) and the soluble fraction, containing recombinant Msf, mixed with Ni-NTA agarose for 1 hour at 4°C. Recombinant Msf regions were eluted with 250 mM imidazole and exhaustively dialysed against 50 mM Tris (pH 7.5) at 4°C. Purified proteins were subject by SDS-PAGE and Western blotting (using anti-Msf polyclonal antibody) to assess purity and the ability to trimerise. Msf_1–422_, _1–203_ and _1–86_ were subject to size exclusion chromatography. To determine the size and oligomeric state of the MSF constructs, the proteins were individually subjected to size exclusion chromatography. In brief, samples were loaded onto a HiLoad 16/60 Superdex 200 (GE Healthcare, UK) equilibrated in 50 mM Tris, pH 8 and 200 mM NaCl and separated based on their size and shape. A molecular mass was determined for each construct by comparison of peak elution volume against known protein standards (Bio-rad, UK).

**Table 2:** Primers used in cloning and site directed mutagenesis.

Primer	Purpose	Sequence 5’-3’
Forward Msf_1_	Cloning/ site directed mutagenesis	GATATACATATGAACAATGAAGAGCAAGAA
Forward Msf_39_	Cloning	GATATACATATGAATTCAGATTGGGCAGTA
Forward Msf_81_	Cloning	GATATACATATGGACCTCACAGATCTGACC
Forward Msf_197_	Cloning	GATATACATATGGAGTTCTTGAGCGCAGATACG
Forward Msf_Δ4–82_	Cloning	GATATACATATGAACAATGAAACAGATCTG
Reverse Msf_86_	Cloning	GTGGTGCTCGAGGGTCAGATCTGTGAGGTC
Reverse Msf_124_	Cloning	GTGGTGCTCGAGGGTGTCGCCGTTCGTCCC
Reverse Msf_203_	Cloning/ site directed mutagenesis	GTGGTGCTCGAGCGTATCTGCGCTCAAGAACTC
Reverse Msf_320_	Cloning	GTGGTGCTCGAGCATAACAGTGATGTTGCC
Reverse Msf_422_	Cloning	GTGGTGCTCGAGCTTGCTGCCGACATTCAA
Forward _K60A_	Site directed mutagenesis	ACAGCCAGAGAAATCACCCTC**GCA**GCCGGCGACAACCTGAAAATC
Forward _KK79-80AA_	Site directed mutagenesis	AACTTCACCTACTCGCTG**GCAGCA**GACCTCACAGATCTGACC
Forward _KIK66-68AIA_	Site directed mutagenesis	CTCAAAGCCGGCGACAACCTG**GCA**ATC**GCA**CAAAACGGCACAAACTTC
Reverse _K60A_	Site directed mutagenesis	GATTTTCAGGTTGTCGCCGGC**TGC**GAGGGTGATTTCTCTGGCTGT
Reverse _KK79-80AA_	Site directed mutagenesis	GGTCAGATCTGTGAGGTC**TGCTGC**CAGCGAGTAGGTGAAGTT
Reverse _KIK66-68AIA_	Site directed mutagenesis	GAAGTTTGTGCCGTTTTG**TGC**GAT**TGC**CAGGTTGTCGCCGGCTTTCAG

*Nde*I and *Xho*I restriction sites are underlined in forward and reverse cloning primers respectively. Nucleotides from the parental gene sequence in the mutagenesis primers are indicated in bold.

### Modelling the interaction between the Msf N-terminal region and the vitronectin binding peptide VA-26

The model of MC58 Msf (identical to H44/76 Msf) has been described previously [30]. The first residue of peptide VA-26 lies within the unstructured twelve residues at the C-terminus of the somatomedin B domain structure of Vn, complexed with plasminogen activator inhibitor-1 (PDB 1OC0; [56]). The peptide is predicted to be 25% β-strand and otherwise unstructured (Jpred3). Hence VA-26 was built as an extended β-strand and relaxed by 10 ps of molecular dynamics followed by 1000 steps of energy minimisation using the conjugate gradient method (Discover 2.98). The electrostatic potentials of both VA-26 and residues 39–82 of the Msf trimer model were calculated using charges appropriate for pH 7 with a Poisson-Boltzmann solver (DelPhi 4.1.1) and mapped to the molecular surfaces and visualised with InsightII-2005 (Accelrys).

### Site directed mutagenesis

One single and two paired amino acid substitutions were introduced into Msf_1–203_ namely K60A, KIK66-68AIA and KK79-80AA. Primer sequences used to generate the changes are listed in Table 2. To introduce the desired nucleotide changes a two-step PCR strategy was used. The first step produced DNA using the Forward Msf_1_ primer paired with the individual reverse mutagenesis primer (Reverse _K60A_, Reverse _KK79-80AA_ or Reverse _KIK66-68AIA_) and the individual forward mutagenesis primers (Forward _K60A_, Forward _KK79-80AA_, or Forward _KIK66-68AIA_) paired with Reverse Msf_203_ primer. The second step used paired PCR products with the desired mutation(s) from the first step as template to produce full length Msf_1–203_ using primers Forward Msf_1_ and Reverse Msf_203_. PCR products were sequenced (www.dnaseq.co.uk) to check the desired mutations had been introduced. PCR produced were subsequently cloned into pET24b and expressed and purified as described above.

### Enzyme linked immunosorbant assay

ELISA plates (96 well, Dynex) were coated overnight with Msf constructs (100 μl of a 3 μM solution) in carbonate buffer pH 9.6. Unbound Msf was removed by washing with ELISA wash (154 mM NaCl containing 0.05% Tween-20). Wells were blocked with StartingBlock (ThermoScientific) for 1 h at room temperature (RT). Msf-coated wells were incubated with either aVn (74 nM) or Biotinylated peptides VA-26 or MV-14/GS-22 (1.5 μM) in Phosphate buffered saline (PBS) for 1h at RT. Unbound protein or peptides were removed by washing with ELISA wash. Bound aVn was detected using rabbit polyclonal anti-vitronectin and anti-rabbit-alkaline phosphatase conjugated secondary antibody (Sigma). Where biotinylated peptides were used, binding was detected using anti-biotin-alkaline phosphatase antibody (Sigma). ELISA plates were developed using SigmaFast p-Nitrophenyl phosphate substrate and the absorbance was measured at 405 nm. When anti-Msf_1–203_ antibodies were used in inhibition studies, wells were pre-incubated with antibody (0–5 μg/ml) for 30 min prior to addition of aVn. Alternatively ELISA plates were coated with aVn (100 μl of a 74 nM solution) and after washing and blocking as above, Msf constructs were added at a concentration of 3μM. Bound Msf constructs were detected using rabbit anti-Msf polyclonal antibodies and anti-rabbit alkaline phosphatase conjugate.

### Serum bactericidal assay (SBA)

Bacteria were diluted in PBS containing Ca^2+^ and Mg^2+^ (PBSB), to obtain *c*. 10^3^ bacteria in a final volume of 100 μl in a microtitre plate. Bacteria were incubated with 10% serum or heat-inactivated serum, in the presence of 0.1–5 μg/ml purified rabbit anti-Msf_1–203_ antibody or anti-Nm polyclonal antiserum (1%) for 60 min. Percent killing was obtained by comparison of colony forming units (cfu) from wells containing serum versus cfu from wells exposed in a similar manner to heat-inactivated serum (56°C, 30 min).

### Statistics and data presentation

Unless otherwise stated, experiments were carried out generally on three separate occasions and triplicate estimations were performed within each experiment. Mean values from at least three independent experiments (*n* = 3) were used to obtain the overall means and standard deviations as stated in each figure legend. Statistical differences between mean values were determined using ANOVA with Tukey’s post-hoc test. Significant *P*-values are indicated on figures as follows * <0.05, ** <0.001 and *** <0.0001.
